# Otoneurologic findings in Type 1 Diabetes mellitus patients

**DOI:** 10.1016/S1808-8694(15)31130-7

**Published:** 2015-10-20

**Authors:** Rafaele Rigon, Angela Garcia Rossi, Pedro Luiz Cóser

**Affiliations:** 1 Speech and hearing therapist (MS student in Human Communication Disorder - Federal University of Santa Maria); 2 Speech and Hearing Therapist; PhD in Human Communication Disorders - Federal University of São Paulo - Paulista School of Medicine (Associate Professor - Department of Otorhinolaryngology/Speech and Hearing Therapy - Federal University of Santa Maria); 3 MD; PhD in Human Communication Disorders - Federal University of São Paulo - Paulista School of Medicine (Assistant Professor of Otorhinolaryngology - Deparment of General Practice Medicine - Federal University of Santa Maria)

**Keywords:** diabetes mellitus, vestibular function tests, dizziness

## Abstract

**Summary:**

Metabolic alterations, as they occur in Diabetes mellitus, have been mentioned in the development and maintenance of complaints related to the vestibular and auditory organs.

**Aim:**

To investigate the vestibular system in Type 1 Diabetic mellitus population.

**Material and method:**

The present study was developed with 19 individuals, being 10 females (52.6%) and 9 males (47.3%), with ages varying from 8 to 25 years old, with medical diagnosis of Type 1 Diabetes mellitus. For result comparison, a control group was selected with others 19 individuals, matching the study group in age and gender. The evaluation protocol encompassed interview, otoscopic inspection, dynamic and static balance evaluation, cerebellar tests and vectoelectronystagmographic evaluation.

**Study design:**

Clinical prospective.

**Results:**

Alteration in the vectoelectronystagmographic evaluation were found in 36.84% (n=7) Type 1 Diabetes mellitus individuals, being 21.06% (n=4) Peripheral Deficiency Vestibular Syndrome and 15.79% (n=3) Peripheral Irritative Vestibular Syndrome.

**Conclusion:**

We conclude that Type 1 Diabetes mellitus individuals can have their vestibular organ affected, even if there are no otoneurologic complaints.

## INTRODUCTION

According to data from the Brazilian Society of Diabetes, there about 151 million diabetic persons in the world today, that is, 4.6% of the world population. In Brazil, Diabetes mellitus affects 7.6% of the population^1^.

Diabetes mellitus is a chronic systemic disease, related to an absolute or relative insulin deficiency, manifested by a deficient insulin secretion by the pancreas and/or a deficient insulin action on the target tissue^2^ and, according to Kuzuya^3^, insulin action deficiency causes chronic hyperglycemia, leading to characteristic abnormalities in the metabolism of carbohydrates, lipids, proteins and others. Rybak^4^ adds that such condition may be associated to vestibular dysfunction.

Glucose metabolism has great influence on the inner ear, and both hypoglycemia and hyperglycemia may affect its normal functioning. Patients with glucose metabolism alteration, as it happens in Diabetes, may present with auditory or vestibular symptoms, or both^5^.

When there are alterations in insulin release by the pancreas, or in its binding to the receptor with secondary glycemia alterations, there is energy availability impairment and consequent inner ear dysfunction^6^.

Because of the high number of individuals with Diabetes mellitus estimated in the Brazilian population, there is a growing concern among health professionals in researching the implications of such pathology, trying to avoid possible secondary disorders caused by Diabetes, which may impact the life quality of these individuals.

Having in mind the close relationship between carbohydrate metabolism disorders and vestibular and/or hearing alterations and the relevant prevalence of these disorders in the general population, this study has its justification, aiming at investigating the vestibular apparatus in a population of type 1 Diabetic patients.

## MATERIALS AND METHODS

The population selected for this study encompassed 29 individuals with medical diagnosis of type I Diabetes mellitus, and the data collection was carried out from April to December of 2005.

Of the 29 individuals selected, 10 were taken off the study: one pregnant female; one male with visual impairment acquired after diabetes onset; one male because of visual and hearing impairment caused by diabetes; and seven other individuals, four females and three males because they did not show up for exams when scheduled to.

After excluding these 10 individuals from the sample of 29 selected for the study, our sample encompassed 19 individuals, 10 females (52.6%) and 9 males (47.3%), with ages ranging between 8 and 25 years.

For result comparison we selected an age and gender matching control group. This group was made of 19 individuals, 10 women and 9 men, with age varying between 8 and 25 years. As exclusion criteria these individuals should not present any hearing or vestibular complaint, as well as medical diagnosis of type I Diabetes mellitus or other diseases that could interfere in the assessment.

All the individuals who participated in this investigation (diabetic and controls), before undergoing the assessment protocol, were informed about the assessment procedure and, agreeing with it, signed an informed consent form (the individual him/herself if adult, or parent/guardian if underage) for the evaluation. This investigation was approved on March, 2005 by the Research Ethics Committee of our institution, under protocol # 005/2005.

The otoneurologic assessment protocol encompassed the medical interview, otoscopic inspection, static and dynamic balance assessment, cerebellar tests and vectoelectronystagmographic evaluation.

Initially, the interview encompassed questions about Diabetes, such as: disease onset time, insulin use, diet control and complications such as hyperglycemia and hypoglycemia spells and other health complications; hearing complaints, vestibular complaints such as dizziness, nausea, vomit, unbalance and headaches.

In order to assess static and dynamic balance and cerebellar function, we carried out the Gait, Romberg, extended arms, Diadochocinesia e Dysmetria (that is index finger - knee) tests, which were first carried out with the eyes open and eyes closed after that. The movements performed were maintained. These are tests of complementary importance because they offer additional topodiagnostic information that adds to other data obtained from the vestibular function exam, and never in isolation.

In order to perform the vestibular test, we used the Computerized Vectoelectronystagmography SCV 5.0 system, proposed by Castagno^7^. Such system encompasses a method of eye movement inscription based on surface electrode capture of electrical potential variations between the cornea (+) and the retina (-) which happens when we move our eyes. It is basically used to record nystagmus, which is the movement most interesting for otoneurology, made up of a set of fast and slow components which happen in turns.

The subjects' skin was cleaned with alcohol in cotton balls in order to effectively capture the electrical potential by means of four electrodes, made up of low polarizing silver, fixed in the periorbital region with electrolytic paste and adhesion tape. The ground electrode (indifferent) was fixed to the frontal region, the upper electrode in the middle line (two centimeters above the glabella) and one electrode at each eye outer corner.

The exam encompasses: eye movement calibration; semi-spontaneous nystagmus (SSN), directional or fixating nystagmus; spontaneous nystagmus (SN) with eyes open and eyes closed; optokinetic nystagmus; pendular tracking; decreasing pendular caloric test (DPCT); and caloric test carried out with warm water (44°C) and cold water (30°C and, if there was no response, we used the water at 18°C, according to Fitzgerald & Hallpike^8^.

The exams traces were analyzed as to the type of calibration, presence of spontaneous and semi-spontaneous nystagmus, optokinetic nystagmus symmetry, type of pendular tracking, symmetry of clockwise and counterclockwise nystagmus beat at DPCT and both quantitative and qualitative analysis of the caloric test.

Qualitative analysis is related to overreactions, when any of the values obtained is greater than 50°/s; underreaction when there is any value below 3°/s; and no reaction when no answer is achieved in the same ear under the temperatures studied (44°C, 30°C and 18°C).

Quantitative analysis is carried out when the results attained from the four stimulations are normal (between 3°/s and 50°/s). In order to compare the values corresponding to the same ear or the same direction of nystagmus, we used Jongkees's formula. The result is said to be normal when this index is lower than 30%; labyrinth preponderance (LP), when both values regarding the same ear are greater than the responses of the contralateral ear; and directional preponderance (DP) when both values are in the opposite direction. LP characterizes a deficit labyrinthine disease (the side with lower post-caloric nystagmus) and DP characterizes an irritative labyrinthine disorder^9^.

It is possible to establish the following lesion locations: peripheral, located in the labyrinth and/or in the VIII nerve, down to where it enters the brain stem; and central, located from the point where the VIII nerve enters the brain stem, in its nuclei via and inter-relations^10^.

For data analysis we compared the findings regarding static and dynamic balance evaluations and the vestibular exam between the group of patients with Type I Diabetes mellitus and the control group; and we also carried out comparisons relating to a complaint of dizziness with alterations prevalence at the Vectoelectronystagmography (VENG). In order to compare Optokinetic values, DPCT and caloric test; we used the Kruskal-Wallis test in order to check the statistically significant differences between the groups.

## RESULTS

In the sample of type I Diabetes mellitus individuals, when they were questioned about dizziness, 42% of the patients said they did not have such complaint. While 47% reported this problem in specific occasions of hypoglycemia, and 11% said they felt dizzy for other reasons.

Table 01 depicts the results of the static and dynamic balance evaluations, together with cerebellar function in both the diabetic and control patients.

**Table 01:** Results from the dynamic and static balance evaluation (Gait, Romberg, Romberg-Barré and Unterberger) and the cerebellar function (Extended Arms, Dysmetria and Diadochocinesia) carried out in the Control and Diabetic groups.

		Control (n=19)	Diabetic (n=19)
	Star	5,26%	10,53%
Gait	Deviation	10,53%	10,53%
	No alteration	84,21%	78,95%
Romberg	Lateropulsion	5,26%	10,53%
	No alteration	94,74%	89,47%
Romberg-Barré	Lateropulsion	21,05%	31,58%
	No alteration	78,95%	68,42%
	Advance	5,26%	31,58%
Unterberger	Deviation	10,53%	5,26%
	No alteration	84,21%	63,16%
	Divergence	0,00%	26,31%
Arms extended	No alteration	100,00%	73,68%
Diadochocinesia	Dysdiadochocinesia	0,00%	0,00%
	No alteration	100,00%	100,00%
	Dysmetria	0,00%	0,00%
Dysmetria	No alteration	100,00%	100,00%

Table 02 depicts the vectoelectronystagmographic results of both the diabetic and control groups.

**Table 02:** Results from the Vectoelectronystagmography carried out in both the Control and Diabetic group.

		Control (n=19)	Diabetic (n=19)
Calibration		Regular (100,00%)	Regular (100,00%)
	Type I	89,47%	78,95%
Tracking	Type II	10,53%	21,05%
NSE		Absent (100,00%)	Absent (100,00%)
NE		Absent (100,00%)	Present (5,26%)
			Absent (94,74%)
Optokinetic		Symmetric (100,00%)	Symmetric (100,00%)
PRPD		Symmetric (100,00%)	Symmetric (100,00%)
	Normal	100,00%	63,16%
Caloric test	PL		21,05%
	PD		15,79%

(Table 03)

**Table 03:** Kruskal-Wallis test results used to compare the right side (D) and left side (E) nystagmus averages in the optokinetic nystagmus study between the Control and Diabetic groups.

Control (n=19)		Diabetic (n=19)		Valor de p
Mean R and L (VACL - °/s)	Standard deviation	Mean R and L (VACL - °/s)	Standard deviation	
6,90%	4,45	6,93%	4,94	0,9883

Test analysis: At the 5% (0.05) significance level, the p value is higher than 0.05; therefore, there is no significant difference between the mean values of optokinetic nystagmus between the control and diabetic groups.

Test analysis: at a 5% significance level (0.05), p is greater than 0.05, therefore, there is no difference between the optokinetic nystagmus test averages between the two groups.

(Table 04)

**Table 04:** Kruskal-Wallis test results used to compare VACL averages of the clockwise and counterclockwise direction nystagmus in the PRPD study between Control and Diabetic groups.

	Control (n=19)		Diabetic (n=19)		
	Mean	Standard deviation	Mean	Standard deviation	Valor de p
VACL (°/s) – clockwise	24,68	8,81	20,89	10,35	0,1249
VACL (°/s) – counterclockwise	22,84	10,31	19,42	8,74	0,4129
Nystagmus directional edominance	7,69%	5,16	6,23%	5,44	0,3725

Test analysis: at the 5% (0.05) significance level, all the p values are higher than 0.05; therefore, there is no significant difference between the VACL mean values in the clockwise and counter-clockwise values and the nystagmus directional predominance, between the control and diabetic groups.

Test analysis: at 5% significance level (0.05), all p values are above 0.05; therefore, there is no significance difference between VACL averages in the clockwise and counterclockwise direction and in the nystagmus directional predominance between the Control and Diabetic groups.

(Table 05)

**Table 05:** Kruskal-Wallis test results used to compare VACL averages obtained from the Caloric Test labyrinthine stimulation in the temperatures of 44°C and 30°C in the right ear (RE) and left ear (LE), between Controls and Diabetics.

	Control (n=19)		Diabetic (n=19)		
	(VACL - °/s) Mean Value	Standard deviation	(VACL - °/s) Mean Value	Standard deviation	Valor de p
44°C OD	15,57	6,98	11,05	8,40	0,0261
44°C OE	17,73	8,31	16,26	12,84	0,2139
30°C OD	18,57	7,41	11,31	6,42	0,0074
30°C OE	20,57	7,38	9,73	5,48	<,0001

Test analysis: at the 5% (0.05) significance level, the p values are higher than 0.05 in the temperatures of 44°C RE, 30°C RE and 30°C LE; therefore, there is a significant difference between the VACL mean values in these temperatures, between the control and diabetic groups.

Test analysis: at the 5% significance level (0.05), p values are greater than 0.05 in the temperatures of 44°C RE, 30°C RE and 30°C LE; therefore, there are significant differences between VACL average values in the above-mentioned temperatures between the Control and Diabetic groups.

In regards to dizziness complaints related to VENG alterations, from he seven individuals who presented test alterations among the diabetic patients, 14.28% (n=1) did not have complaints, 14.28% (n=1) complaint of dizziness for other reasons, and 71.42% (n=5) complained of dizziness in specific hypoglycemic episodes.

## DISCUSSION

Gender-related VENG alterations did not prove to be significant in our sample, since 4 women and 3 men had alterations. In the literature we consulted, there was not a citation of this type of finding in matching age and metabolic disorder population, leading us to believe that there may not be significant differences in regards to vestibular alteration as to gender in this population.

In our sample, nine individuals (47.36%) had dizziness in specific hypoglycemia episodes. Five of these individuals (26.31%) had VENG alterations, and three of these individuals (15.78%) had deficitary peripheral vestibular syndrome and the other two (10.52%) had irritative peripheral irritative syndrome. Sherer & Lobo^11^ also found complaints related to specific hypoglycemic episodes studying a population of 12 type I Diabetes mellitus individuals, and such complaint was present in 25% of the individuals in their sample and, those who reported this type of dizziness episode had Irritative Vestibular Syndrome seen at the vectoelectronystagmography.

Two individuals (10.52%) complained of dizziness related to other causes which were not related to hypo or hyperglycemia, and VENG was found altered in one of these individuals. This individual presented deficitary vestibular syndrome and Diabetes duration time of less than one year.

The other eight individuals did not complain of any sort of dizziness; however, one of these individuals presented VENG alterations and had irritative peripheral vestibular syndrome and one year of diabetes. In a sample of 46 Type I Diabetes mellitus patients, Biurrun^12^ did not find any vestibular symptoms complaint, and 12 individuals showed vectoelectronystagmography alteration (2 individuals with labyrinthine predominance and 10 other individuals with directional preponderance). Rybak4 mentions a high rate of abnormalities seen at the vectoelectronystagmographic results in Diabetes mellitus patients who complained of dizziness.

Comparing the results of static and dynamic balance tests (Gait, Romberg, Romberg-Barré and Unterberger) and cerebellar function (Extended Arms, Dysmetria and Diadochocinesia) between Control and Diabetics groups, no significant difference was found. In the literature reviewed, we did not find reports of these tests being used in this population for comparison purposes.

At the eye movement calibration, pendular tracking, spontaneous and semi-spontaneous nystagmus we did not find major differences between diabetic and control individuals.

Only one individual presented with closed eyes spontaneous nystagmus with 3°/s VACL which may be considered normal according to Ganança^13^ who say that in normal individuals this type of nystagmus may be seen, although rare, with velocities equal to or below 6°/s; and since this has been an isolate finding in this individual's test, and he did not show any alteration in the other tests, it was not considered abnormal.

For the Optokinetic Nystagmus and PRPD tests, we used the Kruskal-Wallis test to check the statistically difference between the groups, and such difference was not found. Gawron^14^ found optokinetic nystagmus alteration in 37.90% of his sample, associating such alteration to the long duration of Diabetes, relating it to a central involvement. Not finding this type of alteration in our sample could be explained by the small size sample with the long time of diabetes duration.

By applying the Kruskal-Wallis test in order to verify this statistically significant difference between VACL value averages obtained with the labyrinthine stimulation by the Caloric Test in the temperatures of 44°C and 30°C in both the right and left ears between controls and diabetics, we found a significant difference in the temperatures os 44°C RE, 30°C RE and 30°C LE, and such difference was more significant in the temperature of 30°C in both ears. VACL value averages were smaller in the diabetic group when compared to the control group. Biurrun12 also found reduced responses to post-caloric nystagmus in diabetic patients, comparing them to the control group, and such difference was statistically significant in the temperatures of 44°C and 30°C in the left ear.

The caloric test was altered in 36.84% (n=7) of the sample, 21.06% (n=4) of those with labyrinthine preponderance, two to the right and two to the left; and 15.79% (n=3) with nystagmus directional preponderance, one to the right and two to the left. The rest of the sample, 63.15% (n=12), had normal results. Biurrun^12^, in a sample with 46 patients with Type I Diabetes mellitus, reported to having found caloric test alterations in the electronystagmography of 26% (n=12) of the patients, 4.33% (n=2) of those with right labyrinthine preponderance and 21.6% (n=10) with directional preponderance, six to the right side and four to the left. Sherer & Lobo^11^ found, in a sample of 12 Type I Diabetes mellitus individuals, normal results for this test in 33.33% of the sample, while 50% (n=6) of the individuals presented directional nystagmus preponderance, and other 16.7% (n=2), showed labyrinthine preponderance without side specification. Gawron^14^, in a sample with 95 individuals, characterized by the same aforementioned metabolic disorder, found labyrinthine preponderance in the caloric test of 4.22% (n=4) individuals and directional preponderance in 7.36% (n=7) of the sample, the side was also not specified.

Through data obtained from the vectoelectronystagmographic evaluation, we found alterations in this test in about 36.84% (n=7) of the sample, 21.06% (n=4) with deficitary peripheral vestibular syndrome and 15.79% (n=3) with irritative peripheral vestibular syndrome. The remaining of the sample, 63.15% (n=12), presented normal results. Biurrun^12^ did not qualify the alterations found in the electronystagmography as vestibular syndromes, only described the type of alterations encountered. Sherer & Lobo11, in a sample with 12 individuals, found normal otoneurological results in 33.33% of the sample, while 50% (n=6) of the individuals had irritative peripheral vestibular syndrome and 16.7% (n=2), had deficitary peripheral vestibular syndrome. Gawron^14^, also did not call vestibular syndromes those alterations found at the electronystagmography, only described the alterations found, saying that most of the alterations found resembled central alterations. We see that our findings agree with those from the consulted literature, although we did find different types of alterations in different proportions when we compare our findings to those aforementioned. Notwithstanding, we could see that there are alterations noticed at the vestibular evaluation of the population studied.

Our findings are in agreement with those from Biurrun^12^, Rybak^4^, Jaurégui-Renaud^15^, Darlington^16^, Perez^17^, Gawron^14^, Gawron^18^, Nicholson^19^ and Sherer & Lobo^11^, who state that there are vestibular alterations, of different types, in Diabetes mellitus individuals.

In closing, we have to highlight that we found very few papers in the literature relating Type I Diabetes mellitus to alterations in the vestibular apparatus, characterizing types of alterations related to the complaint of diziness, as well as duration time of this metabolic disorder, as we did in the present study, although almost all the authors consulted stated that metabolic disorders cause alterations in the vestibular system.

Having seen the findings of the present study, we submit that greater attention should be given to the vestibular apparatus of Type I Diabetes mellitus patients, including otoneurologic investigation in the routine exams of this population, as well as more studies with a larger number of individuals.

## CONCLUSION

Through this study, in which the vestibular apparatus of individuals with Type I Diabetes mellitus was assessed by means of an otoneurological evaluation, we concluded that the perfect functioning of the vestibular apparatus may be impaired in patients affected by this type of Diabetes, even when such individuals do not have complaints.
